# Supplementary material for: Cellular imbalance of specific RNA-binding proteins associates with harmful R-loops
Source: PLoS Genet. 2025 Jul 2;21(7):e1011491. doi: 10.1371/journal.pgen.1011491 (PMC12251259; doi:10.1371/journal.pgen.1011491)
Supplement: S2 Table — (PDF) [file pgen.1011491.s004.pdf]

S2 Table. Yeast strains used in this study.

Strain	Genotype	Source
YDZ7005-16	*MATα ade2-1 leu2-3,112 trp1-1 can1-100 ura3Δ his3-11,15 GALpsi+ ssd1-Δ2 CBP80::ProtA::HIS5*	(1)
YAR015-4C	*MATα ade2-1 leu2-3,112 trp1-1 can1-100 ura3Δ his3-11,15 GALpsi+ ssd1-Δ2 CBP80::ProtA::HIS5 hpr1ΔHIS3*	This study
YBP249	*MATa ade2-1 can1-100 his3-11,15 leu2-3,112 trp1-1 ura3-1 bar1Δ RAD5*	(2)
HPBAR1-R	*MATa leu2-3,112 trp1-1 can1-100 ura3-1 ade2-1 his3-11,15 bar1Δ hpr1ΔHIS3 RAD5*	(3)
HPR1-D2	*MATα ura3-3::ADH1-AtTIR1-9myc (URA3) hpr1::hpr1-aid (hphB) RAD5 bar1Δ*	(3)
SCHY58A	*MATa ade2-1 can1-100 his3-11,15 leu2-3,112 rad5-535 trp1-1 ura3-1 hpr1Δ::KanMX*	(4)
HPR1DGK	*MATa/MATα ade2-1/ade2-1 his3-11,15/ his3-11,15 trp1-1/trp1-1 leu2-3,112/leu2-3,112 can1-100/can1-100 ura3-3::ADH1-AtTIR1-9Myc (URA3)/ ura3-1 hpr1::hpr1-aid (hphB)/hpr1Δ::KanMX4*	This study
BY4741	*MATa his3Δ leu2Δ0 met15Δ ura3Δ0*	Euroscarf
BDIS3-1 (YOL021C)	BY4741 *MATa his3Δ leu2Δ0 met15Δ ura3Δ0 dis3-1*	(5)
BRIE1D (YGR250C)	BY4741 *MATa his3Δ leu2Δ0 met15Δ ura3Δ0 rie1Δ::kanMX4*	(6)
BRIM4D (YHL024W)	BY4741 *MATa his3Δ leu2Δ0 met15Δ ura3Δ0 rim4Δ::kanMX4*	(6)
BSHE2D (YKL130C)	BY4741 *MATa his3Δ leu2Δ0 met15Δ ura3Δ0 she2Δ::kanMX4*	(6)
BRNH1D (YMR234W)	BY4741 *MATa his3Δ leu2Δ0 met15Δ ura3Δ0 rnh1Δ::kanMX4*	(6)
WMC1-1A	*MATa ade2-1 can1-100 his3-11,15 leu2-3,112 rad5-535 trp1-1 ura3-1 mex67-5*	(7)
BRIEGFP (YGR250C)	BY4741 *MATa his3Δ leu2Δ0 met15Δ ura3Δ0 RIE1::yeGFP:: HIS3MX6*	(8)
BSHEGFP (YKL130C)	BY4741 *MATa his3Δ leu2Δ0 met15Δ ura3Δ0 SHE2::yeGFP:: HIS3MX6*	(8)
WRIEHA	*MATa ade2-1 can1-100 his3-11,15 leu2-3,112 trp1-1 ura3-1 RIE1::3xHA bar1Δ RAD5*	This study
WSHEHA	*MATa ade2-1 can1-100 his3-11,15 leu2-3,112 trp1-1 ura3-1 SHE2::3xHA bar1Δ RAD5*	This study
WDISHA	*MATa ade2-1 can1-100 his3-11,15 leu2-3,112 trp1-1 ura3-1 LYS2p::NatMX6-GAL1p DIS3::3xHA bar1Δ RAD5*	This study

1. Oeffinger M, Wei KE, Rogers R, DeGrasse JA, Chait BT, Aitchison JD, et al. Comprehensive analysis of diverse ribonucleoprotein complexes. Nat Methods. 2007;4(11):951-6.
2. Moriel-Carretero M, Aguilera A. A postincision-deficient TFIIH causes replication fork breakage and uncovers alternative Rad51- or Pol32-mediated restart mechanisms. Mol Cell. 2010;37(5):690-701.
3. San Martin-Alonso M, Soler-Oliva ME, Garcia-Rubio M, Garcia-Muse T, Aguilera A. Harmful R-loops are prevented via different cell cycle-specific mechanisms. Nat Commun. 2021;12(1):4451.
4. Chavez S, Aguilera A. The yeast HPR1 gene has a functional role in transcriptional elongation that uncovers a novel source of genome instability. Genes Dev. 1997;11(24):3459-70.
5. Li Z, Vizeacoumar FJ, Bahr S, Li J, Warringer J, Vizeacoumar FS, et al. Systematic exploration of essential yeast gene function with temperature-sensitive mutants. Nat Biotechnol. 2011;29(4):361-7.
6. Winzeler EA, Shoemaker DD, Astromoff A, Liang H, Anderson K, Andre B, et al. Functional characterization of the S. cerevisiae genome by gene deletion and parallel analysis. Science. 1999;285(5429):901-6.
7. Jimeno S, Rondon AG, Luna R, Aguilera A. The yeast THO complex and mRNA export factors link RNA metabolism with transcription and genome instability. EMBO J. 2002;21(13):3526-35.
8. Huh WK, Falvo JV, Gerke LC, Carroll AS, Howson RW, Weissman JS, et al. Global analysis of protein localization in budding yeast. Nature. 2003;425(6959):686-91.
