# Supplementary material for: Cellular imbalance of specific RNA-binding proteins associates with harmful R-loops
Source: PLoS Genet. 2025 Jul 2;21(7):e1011491. doi: 10.1371/journal.pgen.1011491 (PMC12251259; doi:10.1371/journal.pgen.1011491)
Supplement: S3 Table — (PDF) [file pgen.1011491.s005.pdf]

S3 Table. Plasmids used in this study.

Name	Description	Source
pYES2	Multicopy expression vector with *GAL1* promoter with 2μ origin and *URA3* marker.	Thermofisher
pYES-NPL3	*NPL3* ORF from *S. cerevisiae* cloned into XhoI site of pYES2.	This study
pYES-RIE1	*RIE1* ORF from *S. cerevisiae* cloned into KpnI/EcoRI site of pYES2.	This study
pYES-RIM4	*RIM4* ORF from *S. cerevisiae* cloned into KpnI/SacI site of pYES2.	This study
pYES-RIM4Δ448-718	*RIM4* ORF with aminoacids 448 to 718 deleted cloned into pYES2.	This study
pYES-SBP1	*SBP1* ORF from *S. cerevisiae* cloned into HindIII/XbaI site of pYES2.	This study
pYES-SHE2	*SHE2* ORF from *S. cerevisiae* cloned into XhoI site of pYES2.	This study
pYES-YRA1Δi	*YRA1* ORF without intron from *S. cerevisiae* cloned into BamHI site of pYES2.	This study
pYES-YFP	*YFP* ORF cloned into PvuII/EcoRI site of pYES2.	This study
pYES-RIE1YFP	*RIE1* ORF fused to *YFP* cloned into KpnI/EcoRI site of pYES2.	This study
pYES-SHE2YFP	*SHE2* ORF fused to *YFP* cloned into KpnI/EcoRI site of pYES2.	This study
pYES-RIE1HA	*RIE1* ORF fused to 3xHA tag cloned into pYES2.	This study
pYES-SHE2HA	*SHE2* ORF fused to 3xHA tag cloned into pYES2.	This study
pYES-SBP1-HA	*SBP1* ORF fused to 3xHA tag cloned into pYES2	This study
pYES-RIM4-HA	*RIM4* ORF fused to 3xHA tag cloned into pYES2	This study
pYES-RIM4Δ448-718-HA	*RIM4* ORF with aminoacids 448 to 718 deleted fused to 3xHA tag cloned into pYES2	This study
YEp351	Multicopy vector with 2μ origin and *LEU2* gene as marker.	(1)
YEpDIS3	*DIS3* ORF plus 457pb upstream and 126pb downstream from *S. cerevisiae* cloned into SacI/SphI site of YEp351.	This study
YEpDIS3-HA	*3-HA* epitope cloned at the C-terminal part of *DIS3* in the YEpDIS3 plasmid	This study
YEpYRA1Δi	*YRA1* ORF without intron plus 500pb upstream and downstream from *S. cerevisiae* cloned into BamHI site of YEp351.	This study
MW90	Yeast genomic DNA library constructed in YEp351.	(2)
MW90-C1	MW90 plasmid containing the fragment from position 280678 to 288971 of the chr. XV of the *S. cerevisiae* genome.	This study
MW90-C1A	MW90-C1 digested with XbaI restriction enzyme.	This study
MW90-C1B	MW90-C1 digested with BamHI restriction enzyme.	This study
MW90-C2	MW90 plasmid containing the fragment from position 987942 to 995171 of the chr. VII of the *S. cerevisiae* genome.	This study
pWJ1213	YCp plasmid containing the Rad52::YFP fusion and *HIS3* marker.	(3)
pWJ1344	YCp plasmid containing the Rad52::YFP fusion and *LEU2* marker.	(3)
pRS413	YCp vector with *GAL1* promoter and *HIS3* marker.	(4)
pRS313-GALRNH1	YCp pRS413 containing the *GALp::RNH1* fusion.	(5)

pRS416	YCp vector with *GAL1* promoter and *URA3* marker.	(4)
pGALRH1	YCp pRS416 containing the *GALp::RNH1* fusion.	R. J. Crouch
pFA6a-3HA-kanMX6	pFA6a plasmid containing a cassette with 3 copies of influenzavirus hemagglutinin (HA) epitope and kanMX6 selectable marker.	(6)
pRS413-GAL::YRA1Δi	Ycp pRS413 containing the GALp::YRA1Δi fusion.	(7)

1. Hill JE, Myers AM, Koerner TJ, Tzagoloff A. Yeast/E. coli shuttle vectors with multiple unique restriction sites. Yeast. 1986;2(3):163-7.
2. Waldherr M, Ragnini A, Jank B, Teply R, Wiesenberger G, Schweyen RJ. A multitude of suppressors of group II intron-splicing defects in yeast. Curr Genet. 1993;24(4):301-6.
3. Lisby M, Rothstein R, Mortensen UH. Rad52 forms DNA repair and recombination centers during S phase. Proc Natl Acad Sci U S A. 2001;98(15):8276-82.
4. Christianson TW, Sikorski RS, Dante M, Shero JH, Hieter P. Multifunctional yeast high-copy-number shuttle vectors. Gene. 1992;110(1):119-22.
5. Garcia-Benitez F, Gaillard H, Aguilera A. Physical proximity of chromatin to nuclear pores prevents harmful R loop accumulation contributing to maintain genome stability. Proc Natl Acad Sci U S A. 2017;114(41):10942-7.
6. Bahler J, Wu JQ, Longtine MS, Shah NG, McKenzie A, 3rd, Steever AB, et al. Heterologous modules for efficient and versatile PCR-based gene targeting in Schizosaccharomyces pombe. Yeast. 1998;14(10):943-51.
7. Gavalda S, Santos-Pereira JM, Garcia-Rubio ML, Luna R, Aguilera A. Excess of Yra1 RNA-Binding Factor Causes Transcription-Dependent Genome Instability, Replication Impairment and Telomere Shortening. PLoS Genet. 2016;12(4):e1005966.
