# Supplementary material for: Cellular imbalance of specific RNA-binding proteins associates with harmful R-loops
Source: PLoS Genet. 2025 Jul 2;21(7):e1011491. doi: 10.1371/journal.pgen.1011491 (PMC12251259; doi:10.1371/journal.pgen.1011491)
Supplement: S4 Table — (PDF) [file pgen.1011491.s006.pdf]

S4 Table. Primers used in this study.

Name	Sequence (5’ to 3’)	Use
GCN4 3' F	TTGTGCCCGAATCCAGTGA	qPCR
GCN4 3' R	TGGCGGCTTCAGTGTTTCTA	qPCR
18S_qPCR F	GGAATCGAACCCTTATTCCC	qPCR
18S_qPCR R	TCAACTTTCGATGGTAGGAT	qPCR
NLR021w fw	GGGTCGGGATTTGAGTAGCG	qPCR
NLR021w rv	CTTCTTCCCTTCTGCAGAGG	qPCR
NEL025c PP3 fw	CTCATAAACAGGAAAGACGC	qPCR
NEL025c PP3 rv	ATCTGACCAGGTAGTTC	qPCR
NHR027c fw	AATAAATTCTAGCGTAGTTC	qPCR
NHR027c rv	TCATACTGTACTTGCACCTC	qPCR
ASH1_E1 fw	CATTGGTGTAAGGATACAAACTATC	qPCR
ASH1_E1 rv	TTTTGATTATTAGTTAAGTTGGGTATAC	qPCR
EAR1 fw	GATTCCTTACCTATGCTACCACCA	qPCR
EAR1 rv	TGCATGCTGCTTTCGTAATCATC	qPCR
IST2 CT fw	AGTGGCTACTGAACAAACAAAA	qPCR
IST2 CT rv	GCATCACGATGGCGGTGGTGGTGA	qPCR
HXT1 up	AGCTGGCAGAATCGACGAA	qPCR
HXT1 down	GGTCAGGTGGGCATTTGTTAA	qPCR
5.8S fw	CTTTCAACAACGGATCTCTTGG	Northern blot probe
5.8S rv	GACGCTCAAACAGGCATGC	Northern blot probe
RIE1-HA fw	GGCAAACGGTAGTAATGAAGAGGAAGAATTTTCTAGTG GTGATTATTCTATGGACTACCGGATCCCCGGGTTAATTAA	Cloning
RIE1-HA rv	CCAACTAATACATGAAGAAAAAAAAAGCAGACAAAAACAT TTTATGGACCTGATGCATGAATTCGAGCTCGTTTAAAC	Cloning
SHE2-HA fw	GACGAAGAATTTGATGTTGTCCGCTACTAAATGGCATG ACAAATTTGGTAAATTGAAAAACCGGATCCCCGGGTTAA	Cloning
SHE2-HA rv	GTATATATATATGTTCTATTAACTAGTGGTACTTATTTGCTC TTTTTGAGCTAAAAACTTGAATTCGAGCTCGTTTAAAC	Cloning
RIM4-HA fw	GACATAAATGAAGATAACAAAGCTTATAGTTTAGATTACTACCCAT ACGATGTTCCTGAC	Cloning
RIM4-HA rv	CGTGAATGTAAGCGTGACATAACTAATTACATGATGCTTAGAAGT GGCGCGCCTCAG	Cloning
RIM4Δ-HA rv	TGGTAAGAACGGTGGCAGCAACTTTAACCATCATCAGTTTTACCC ATACGATGTTCCTGAC	Cloning
DIS3Sal fw	GAATATGTCCAATTTCGTTGGAATCGTCC	Cloning
DIS3-HA rv	GTAAACGCCAGGGTTTTCCCAGTCACGACGTTGTAAAACGAC GGTAGAGGTGTGGTCAATAAGAGCG	Cloning
SBP1-HA fw	CTGTAATACGACTCACTATAGGGATGTCTGCTGAAATTGAAGAAG	Cloning

SBP1-HA rv	GGTAAAAGATGTTAATTAACCCGGGGATCCGTTCTTGCTTTTCTTC AGAACC	Cloning
RECOMB rv	GATGTGGGGGGAGGGCGTGAATGTAAGCGTGACATAACT AATTACATGAGAAGTGGCGCGCCTCAGCACTG	Cloning
NPL3-XhoI fw	CGCTAAAACCTCGAGGATAATGTCTG	Cloning
NPL3-XhoI rv	CTCAACTATCTCGAGGGCTTACCTG	Cloning
RIE1-KpnI fw	CCGCATCAGAGGTACCGAGGATG	Cloning
RIE1-EcoRI rv	TTATGGACCTGAATTCATCTAGTAGTCC	Cloning
RIM4-KpnI fw	AGCTTGTGGTACCATGAAAACCG	Cloning
RIM4-SacI rv	CATTCTTTTGGAGCTCTTACGACCAATC	Cloning
SBP1-HindIII fw	GAGAAGAAGTTTCCCAAGCTTGAAAGAAGAAAACCCTCA	Cloning
SBP1-XbaI rv	CAAAACTCTAGCAAAATCTAGAGTTAGAAATAGGGATGT	Cloning
SHE2-XhoI fw	CATAATACCAGACACTCGAGAATGAGCAAAGAC	Cloning
SHE2-XhoI rv	GAGCTAAAAACTCGAGGCCTCAGTTTTTC	Cloning
YFP fw	ATGAGTAAAGGAGAAGAACTTTTC	Cloning
YFP-EcoRI rv	GAGTAACTAGAGGAATTCGGAGTAAT	Cloning
RIE1-KpnI fw	CCGCATCAGAGGTACCGAGGATG	Cloning
RIE1-PH rv	CTCCAGTGAAAAGTTCTTCTCCTTTACTCATCGCTGCGG CAGCGTAGTCCATAGAATAATCACCAC	Cloning
SHE2-KpnI fw	CCTTTTGCATGGTACCAGACACTTAAAAATG	Cloning
SHE2-PH rv	CTCCAGTGAAAAGTTCTTCTCCTTTACTCATCGCTGCG GCAGCGTTTTTCAATTTACCAAATTTGTCATG	Cloning
RIE1comp fw	GCAGAATCCTGCAGCAAATC	Cloning
SHE2comp fw	GATGGTAAGCTGAGCGCCTTG	Cloning
HAtag rv	GATGTGGGGGGAGGGCGTGAATGTAAGCGTGACATAA CTAATTACATGAGAAGTGGCGCGCCTCAGCACTG	Cloning
DIS3-SacI fw	GATACACCCCCAGAGCTCCAAGGTTAGACTACTACAGC	Cloning
DIS3-SphI rv	CTAAATAGTGCATGCCACTCTACAAGAGATATCACG	Cloning
SBP1-3’ fw	ACGTCCCATTCAAAGCTACCA	qPCR
SBP1-3’ rv	TCGGCGTCAGTACCGAAAA	qPCR
SHE2-3’ fw	TAGGCAGCAACCTGCTAGATTTAG	qPCR
SHE2-3’ rv	CGTCCTCATCTGCGCATTT	qPCR
NPL3-3’ fw	CCTAGAGGTGGTTACGATAGTCCAAGAG	qPCR
NPL3-3’ rv	GTTCTGTATGCATCTCTTGGAGGACC	qPCR
RIM4-3’ fw	AGGTTCTGCTGGAAATCACCAT	qPCR
RIM4-3’ rv	AAGGCGGAGGTGGTGACA	qPCR
