# Supplementary material for: Cellular imbalance of specific RNA-binding proteins associates with harmful R-loops
Source: PLoS Genet. 2025 Jul 2;21(7):e1011491. doi: 10.1371/journal.pgen.1011491 (PMC12251259; doi:10.1371/journal.pgen.1011491)
Supplement: S5 Table — (PDF) [file pgen.1011491.s007.pdf]

S5 Table. Antibodies used in this study.

Name	Use	Source	Identifier
S9.6 antibody	IF (1:300)<br>DRIP (6 µg)	Homemade	
HA tag antibody - ChIP Grade	ChIP (10 µg)<br>WB 1:5000	Abcam	Cat# ab9110,<br>RRID:AB_307019
Anti-PKG1 monoclonal antibody	WB 1:10000	Invitrogen	Cat# ab459250<br>Clone22C5D8
Goat anti-mouse IgG peroxidase antibody	WB 1:5000	Merck	A4416
Goat anti-rabbit IgG peroxidase antibody	WB 1:5000	Merck	A6154
Chicken anti-mouse IgG (H+L) antibody, Alexa Fluor 594 conjugated	IF (1:1000)	Invitrogen	Cat# A-21201,<br>RRID:AB_141630
